# Life-history trade-offs and reproductive strategies in Tasmanian devils following disease-induced population decline

**DOI:** 10.1098/rspb.2025.0697

**Published:** 2025-05-21

**Authors:** Kasha Strickland, Menna Jones, Shelly Lachish, Sebastien Comte, Rodrigo Hamede Ross, Paul A. Hohenlohe, Hamish McCallum, Andrew Storfer, Loeske E. B. Kruuk

**Affiliations:** ^1^ Institute of Ecology and Evolution, School of Biological Sciences, University of Edinburgh, Edinburgh EH9 3FL, UK; ^2^ School of Natural Sciences, University of Tasmania, Hobart, Tasmania 7005, Australia; ^3^ Public Health Intelligence Branch, Queensland Public Health and Scientific Services, Queensland Health, Herston, Queensland 4600, Australia; ^4^ Vertebrate Pest Research Unit, NSW Department of Primary Industries, Sydney, New South Wales 2800, Australia; ^5^ Department of Biological Sciences, University of Idaho, Moscow, Idaho 83844, USA; ^6^ Environmental Futures Research Institute, Griffith University, Nathan, Queensland 4111, Australia; ^7^ School of Biological Sciences, Washington State University, Pullman, WA 99163, USA

**Keywords:** life-history traits, precocial breeding, inbreeding depression, fitness, breeding success

## Abstract

Life-history trade-offs can mediate population declines following perturbations, and early reproduction should be favoured when adult survival is impacted more than juvenile survival. In Tasmanian devils (*Sarcophilus harrisii*), following the emergence of a transmissible cancer that caused steep population declines, females started to breed precocially (i.e. at age 1 instead of 2 years old). Here, using 18 years of mark–recapture data from a site where the disease was present (Freycinet Peninsula, Tasmania, Australia), we tested whether: (i) the probability of 1-yea-old females breeding continued to increase over time; (ii) there was a relationship between body size and breeding success for either 1-year-old or adult females; and (iii) there was inbreeding depression in breeding success for either age category. We show that the probability of 1-year-old females breeding did not increase between 2003 and 2021, and that the proportion of precocially breeding females remains at around 40%. We also show that there was no effect of skeletal body size on the probability of breeding, but heavier females were always more likely to breed. Finally, we found no evidence for inbreeding depression in breeding success. We discuss our results in the context of possible constraints by way of limitations to growth in the offspring of precocially breeding females.

## 1. Introduction

Life-history theory states that trade-offs between key fitness traits such as growth, survival and reproduction are central to phenotypic evolution [1]. In particular, resource limitations experienced by wild populations result in a need for individuals to differentially allocate energy to competing life-history functions, which in turn shapes trait evolution [2,3]. Given that the resultant trade-off is often associated with shifts in allocation to different vital rates (i.e. survival versus reproduction), life-history strategies adopted by populations can have important effects on population dynamics [4–6]. Consequently, shifts in life-history traits can be one of the first responses to population perturbances (either via plasticity or evolution) and, in some cases, can mitigate their impacts on population decline [7–9]. For instance, it has been shown that maturation and the timing of reproduction may become earlier following population declines caused by shifts in predation [10–12], over-fishing [13,14] or disease outbreaks [15,16].

While shifts in life-history traits may buffer the effect of extrinsic drivers of population decline [17], another important impact of population decline is the risk of inbreeding depression [18], which can vary in strength across the lifespan [19]. Inbreeding depression describes a decline in fitness-related traits caused by the expression of deleterious recessive alleles as a result of increased homozygosity resulting from mating between relatives [20]. The potential for inbreeding generally increases in declining populations [21] and, coupled with the associated reduction in genetic diversity [22], the resulting inbreeding depression may exacerbate population declines. Furthermore, the strength of inbreeding depression may vary with age [19,23,24]. For instance, the mutation accumulation hypothesis suggests that inbreeding depression may be stronger in later life because selection fails to purge deleterious alleles that are expressed in later life [25]. Alternatively, increased mortality of inbred individuals at early life stages may result in decreased inbreeding depression in later life stages [24]. It is therefore important to consider how inbreeding depression differentially affects life stages when studying how life-history traits might shift following perturbations to populations.

One important example of a life-history shift following population decline has been in Tasmanian devils (*Sarcophilus harrisii*), where females started to breed earlier following the emergence of a fatal, transmissible cancer [15]. Devil facial tumour disease (DFTD) has caused declines of up to 80% across the geographic range of Tasmanian devils, which are the largest carnivorous marsupial endemic to Tasmania, Australia [26,27]. Prior to the emergence of DFTD, almost all female devils reached sexual maturation and started to breed at 2 years of age [15,28]. However, in the 2 years immediately after the emergence of the disease, approximately 40% of females began to breed at 1 year of age [15]. Although the causes of this change remain unknown, such ‘precocial’ breeding may have arisen owing to increased mortality risk or owing to increased food availability associated with the decreasing population sizes, which could have relaxed the trade-off associated with growing to the threshold size to enable sexual maturity. However, it has also been suggested that the offspring of precocial breeders may not be able to breed precocially. This is because females that breed precocially normally give birth at about 14 months, which is generally later in the mating season (May versus February–March) than individuals breeding at 2 or more years old. Therefore, their offspring are unlikely to reach sexual maturity by May of the following year owing to being younger and therefore smaller [28]. Consequently, constraints on continued increases in rates of precocial breeding may arise [29].

In this study, we aimed to follow up on previous studies that showed evidence of a rise in precocial breeding in females post-DFTD emergence [15]. We analysed data from the 18 years after the disease emerged, collected as part of a long-term mark–recapture study of Tasmanian devils on the Freycinet peninsula, Tasmania. Specifically, we tested: (i) whether there was a continued temporal trend showing a further increase in rates of precocial breeding through time; (ii) whether there was evidence for a relationship between age at breeding and body size (measured as both body weight (kg) and head width (mm)); and (iii) for evidence of inbreeding depression in annual breeding success for either 1-year-olds or for adults (i.e. at least 2 years old).

## 2. Material and methods

### (a) Tasmanian devil study site, trapping and phenotypic data

We used mark–recapture data collected on Tasmanian devils from Freycinet Peninsula, Tasmania, Australia between January 1999 and May 2021. We selected to use data from Freycinet rather than other populations where similar monitoring has occurred because the dataset is larger than from other populations, and there is sufficient genetic data to estimate genetic measures of inbreeding (see below). DFTD first appeared at this site in 2001 and has spread rapidly since then, causing population declines of up to 80% [26]. Tasmanian devils were trapped across the entire peninsula up to four times a year using custom-built baited traps [30], with trapping periods timed to coincide with key stages in the breeding cycle: autumn (March/April), early pouch young; winter (June/July), late pouch young; spring (September/October), females lactating with young in dens; summer (December/January), dependent young emerging from dens before weaning in early February. During their first capture, devils were sexed, individually tagged with a microchip and a 3 mm biopsy sample of tissue was taken from the outer edge of the ear for genetic analysis (see below). At first capture and then at all subsequent recaptures, their age, head width (mm; precise linear measure of body size taken across the jugal arch) and body mass (kg) were recorded as described in [30]. Individuals were aged using a combination of head width, molar eruption, molar tooth wear and canine over-eruption [31] and given a birthdate of 1 April for a given year as per Lachish *et al.* [30]. This method of ageing is accurate up to 2 years of age, but most (74%) individuals were first trapped as juveniles and are therefore of known age. Disease status (presence/absence) was determined for each capture by visual inspection for tumours and/or histopathological examination of tumour biopsies [32].

We used a subset of these data that included repeated observations of females of known age, and where size and reproductive status had been recorded. For each observation of each female, their reproductive status was determined using the following criteria: presence of active (lactating) teats, presence of pouch young and/or pouch appearance (e.g. size, intense reddening and secretory activity, all of which have been shown to accurately predict reproductive status in Tasmanian devils) [33]. Using these criteria, we identified whether or not a female had bred or not within that year (defined as April–March), which we hereafter refer to as ‘annual breeding success’ and was recorded as a binary variable. Multiple observations per female in a given year were reduced to a single estimate of breeding success, and in cases where breeding status varied among observations, we retained the first observation that confirmed breeding.

We subset the data to include observations of individuals using a minimum age of 14 months. This was done (i) in order to reduce the conflation between age and size measurements, and (ii) because 14 months is the youngest age at which females have bred in the dataset. Re-analysing the data using an age of 18 months as a threshold did not change the qualitative inferences of the results, but did reduce the dataset such that confidence in parameter estimates decreased. Therefore, we retained the 14 months minimum age cut-off. Age was binned into two age categories for all analyses: 1-year-olds (14–23 months old) and adults (24 months old or older). Because there were very few observations of 1-year-old breeding females prior to 2003, we removed those years from further analyses.

### (b) DNA extraction and genotyping

We extracted DNA from tissue samples using DNeasy kits (Qiagen), and genotyping was done as previously described in [34]. Briefly, single-nucleotide polymorphism (SNP) genotyping was achieved via single-digest *RADcapture* (i.e. ‘Rapture’ [35]) of DNA extracted from tissue. All raw reads from sequencing were first aligned to the *S. harrisii* reference genome [36]. PCR duplicates were removed, and SNP calling was conducted using *gtstacks* [37] on the merged ‘bam’ files from reads generated from two rounds of sequencing (which was done to achieve sufficient read depth [34]). The function *populations* was then used to keep one random SNP per RAD locus and per 10 kb window, to exclude SNPs with a minor allele frequency (MAF) below 1%, to remove individuals with more than 70% missing data and to remove SNPs present in less than 50% of the samples. We then further filtered genotype calls with a read depth of <4 to increase genotyping accuracy, before reapplying the filtering parameters explained above. This resulted in a total of 2105 SNPs genotyped in a total of 584 individuals for the whole study population (which was further restricted for use with phenotypic data in our analyses—see below).

### (c) Inbreeding coefficients

We measured variation in inbreeding using genomic inbreeding coefficients that were estimated in GCTA [38]. We used $\hat{F}$III (hereafter *F*_GRM_), which estimates the allelic correlation between gametes, because it is closely correlated with runs of homozygosity on the genome (*F*_ROH_) and is therefore a reliable measure of the genomic consequences of inbreeding [38]. We ensured that *F*_GRM_ measures were robust to SNP filtering by varying the MAF cut-off criterion at 1%, 5% and 10%. *F*_GRM_ estimates were all very highly correlated, regardless of which MAF cut-off we used (*r* > 0.99%). *F*_GRM_ ranged from −0.37 (indicating outbreeding) to 0.36 (indicating significant inbreeding; median *F*_GRM_ = −0.04, variance = 0.006).

### (d) Statistical analyses

To test our objectives, we ran two generalized linear mixed effects models using a Bayesian framework in Stan using the *brms* R package [39]. The response variable for both of these models was annual breeding success (see description above) which was fit as a binary variable, and was therefore fit as a logistic regression with a logit link via the Bernoulli family.

The first model we ran was used to (i) determine whether there was a temporal trend in the probability of precocial breeding; (ii) estimate the relationship between body size (measured as body weight (kg) and head width (mm)) and annual breeding success; and (iii) estimate whether the relationship between body size and annual breeding success differed between 1-year-olds and adults. This model included the following variables as fixed effects: calendar year of capture, age (defined as a category with two levels: ‘1-year-olds’ or ‘adults’), head width (mm), body weight (kg) and DFTD status (present or absent). DFTD status was included because it may affect breeding success [28]. To test whether the probability of a 1-year-old breeding increased through time, we fit the interaction between calendar year and age. This interaction term estimated the difference in the temporal change in annual breeding success for adults and 1 year olds, allowing us to test if there was temporal change in annual breeding success for 1-year-olds, but not in adults, or *vice versa*. We also modelled the interaction between body size measurements (head width and body weight) and age to determine whether the effect of size was different for the different age categories. For instance, this allowed us to test whether larger 1-year-old females were more likely to breed than smaller ones. Although head width and body weight are both measures of body size, and are moderately correlated (*r*^2^ = 0.55), they reflect different key aspects of the trait: head width reflects overall skeletal size, whereas body weight includes important aspects of body condition [40]. As a result, we fit both variables in the model to test if and how these different (albeit related) aspects of body size might be impacting breeding success. Year, individual ID, trap ID (i.e. the trap number in which the individual was caught, the locations of which are the same throughout the study period) and calendar month were all included as random effects to account for nonlinear temporal changes in annual breeding success, repeated measures of annual breeding success for females, the effect of spatial heterogeneity and seasonality of breeding displayed by the species. After the data selection process described above, this model was fit with a dataset that contained a total of 372 observations of 253 females caught between 2003 and 2021 (*n* = 195 observations of 195 1-year-olds and *n* = 177 observations of 130 adult females).

The logistic regression described above fits the effect of time on annual breeding success as a sigmoid-shaped curve. To explore whether there was evidence for further nonlinearity in the effect of year on annual breeding success for either 1-year-olds or adults, we refit the model described above to instead fit year as a smoothed term with five knots. This smoothed effect of year was fit for each age category, resulting in an estimate of the smoothed effect of year for both 1-year-olds and for adult females.

The second model we ran was used to investigate whether there was evidence for inbreeding depression in annual breeding success. This model also fit annual breeding success as a binary response variable, and included age (as a two-level category ‘1-year-olds’ or ‘Adults’), head width, weight and *F*_GRM_ as fixed effects. We further included the interaction between age and *F*_GRM_ to determine whether inbreeding depression was stronger in one age category than the other. As above, year, ID, trap ID and calendar month were included as random effects. To help reduce model complexity, we did not run this model with a linear effect of year (and therefore the interaction between age and year) because there was little/no evidence for this term (see §3) and also this effect was not the main focus of this model as we were primarily interested here in testing for inbreeding depression. This model was run with a smaller dataset that included females who had phenotypic observations of annual breeding success as well as genetic data from which we could estimate *F*_GRM_ (see description above). This dataset therefore included *N*_observations_ = 126 from *N*_females_ = 85 (*n* = 62 observations of 62 1-year-olds and *n* = 64 observations of 48 adult females).

Both models were fit with normal priors with 5 s.d. on the fixed effects and half-Cauchy priors with 3 d.f. on the random effects. Both models were run for 9000 iterations with a warmup period of 2000 runs across four chains, and convergence was assessed by ensuring R-hat was below 1.01, effective sample sizes for all parameters were at least 1000 and by visually ensuring chains had mixed well.

## 3. Results

### (a) Temporal trend in precocial breeding

**Figure 1.**
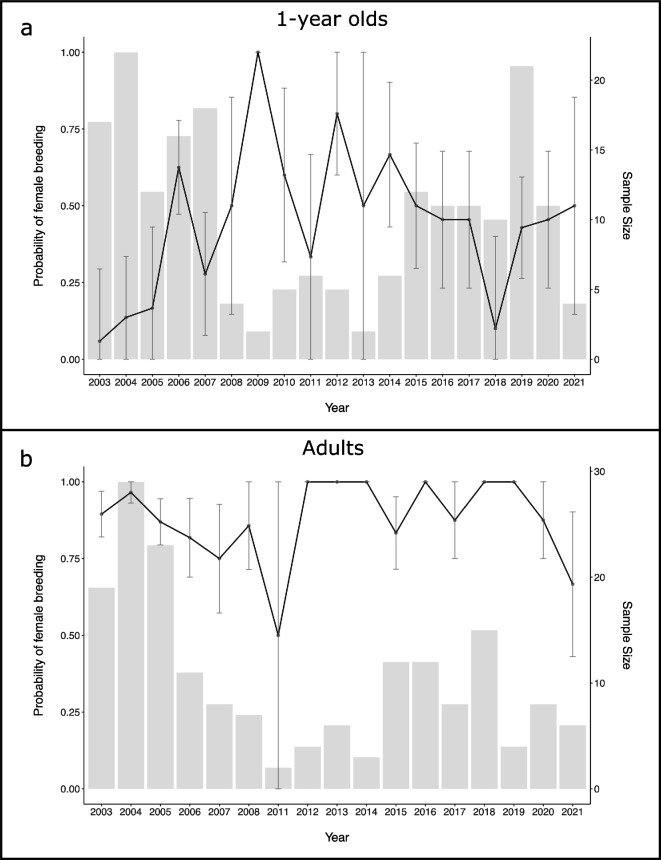
Plot showing the raw data for the number of females caught in each year (grey bars) and the probability that those females would have bred (black line) for each year between 2003 and 2021 for 1-year-old females (a) and adult females (b). Lines represents the yearly average probability, with standard errors on that mean included as error bars.

**Figure 2.**
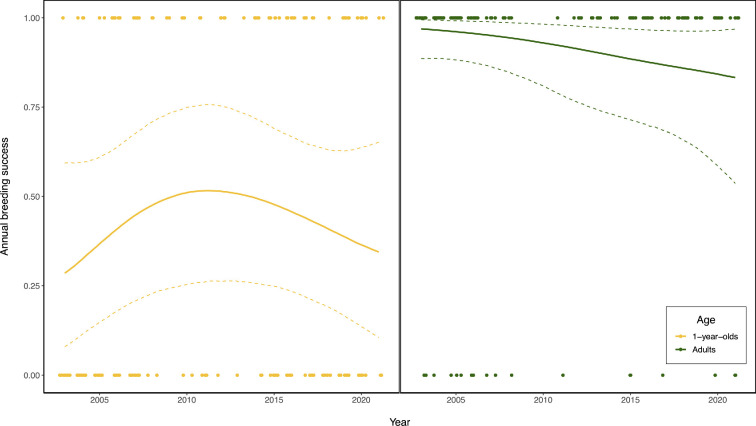
Plot showing the relationship between year and annual breeding success for 1-year-olds (gold) and adults (green). Points show observed data, and regression lines show the predicted relationship between year and annual breeding success derived from a mixed effects model that fits annual breeding success as a function of year, which was fit as a smoothed term with five knots (see §2d for full model structure). Solid line shows predictions derived from the median of the posterior and the dotted lines show upper and lower 95% confidence intervals from posterior distribution.

We did not find any statistical support suggesting that annual breeding success changed through time for either adults or 1-year-olds (table 1, figures 1 and 2). In the raw data, there did appear to be substantial fluctuations through time in the mean probability that a 1-year-old female would breed; however, the standard errors of estimated yearly means of the probability of a female breeding were very large (figure 1). Moreover, we did not find statistical evidence that the probability of a female breeding either increased or decreased linearly between 2003 and 2021, as the posterior distribution for the effect of year was fairly normally distributed and was centred on zero (table 1 and electronic supplementary material, figure S2). That is, the credible intervals of the posterior distribution for the slope estimate of year on probability of breeding were wide and overlapped zero (table 1 and electronic supplementary material, figure S2). In our model, the reference age category was 1-year-olds, and therefore the effect of year reflects the estimated trend for 1-year-olds (table 1). We did find evidence for an interaction term between year and age, suggesting that the estimated effect of year was more positive for adults than for 1-year-olds (table 1). However, while different from the effect for 1-year-olds, the effect of year on breeding success for adults suggested no actual change through time (figure 2). As a result, the data do not support the hypothesis that the probability of a 1-year-old or adult female breeding increased through time.

**Table 1.** Table summarizing results from a generalized mixed effects model used to estimate the effect of body size (weight and head width) and time (year) on annual breeding success. The interaction between age and year, head width and weight were also fit as additional fixed effects. The model was run as a logistic regression given annual breeding success is a binary variable, and therefore all parameters are presented on the logit scale. Posterior medians of linear coefficient estimate for fixed effects and standard deviations for random effects are presented with 95% credible intervals of posterior distribution in parentheses. Full posteriors can be found in the supplementary material.

fixed effects	age_ADULT_	0.12 (−9.52 to 9.94)
	year	0.14 (−0.50 to 1.00)
	head width	0.39 (−0.37 to 1.62)
	weight	6.84 (2.02 to 13.53)
	DFTD	2.39 (−3.33 to 8.55)
	age_ADULT_: year	0.08 (0.01 to 0.20)
	age_ADULT_: head width	−1.46 (−3.79 to −0.15)
	age_ADULT_: weight	1.98 (−2.40 to 7.66)
Random effects	ID	12.83 (3.18 to 28.16)
	trapID	8.23 (1.68 to 18.86)
	year	3.08 (0.18 to 8.71)
	month	7.73 (1.08 to 20.98)

Age was fit as a two-level factor (‘Adult’ or ‘1-year-old’) where the reference level was 1-year-old females; year was fit as calendar year of capture as a linear effect; head width (mm) and weight were fit as linear effects; DFTD was a binary variable describing whether or not the female had DFTD at time of capture (1/0 = yes/no); ID was the unique identifier of each female and accounted for repeated measures of females; TrapID was the unique identifier of each location and accounted for spatial environmental variation; random effect of year accounted for nonlinear changes in probability of breeding; month accounted for seasonality in breeding.

We also did not find statistical support for an effect of year as a smoothed term, suggesting that there was no statistical support for temporally fluctuating changes in the probability of breeding for either age category (table 1, figure 2 and electronic supplementary material, table S1). Fitting year as a smoothed term in the model did appear to capture the initial increase in breeding success in 1-year-olds reported previously [15], however statistical confidence in the smoothed term was very low and the credible intervals of the posterior distribution of the predicted effect of year included zero (table 1, figure 2 and electronic supplementary material, figure S2). As such, our data also do not support the hypothesis that there were nonlinear changes through time in the probability that either a 1-year-old or adult female would breed in a given year.

### (b) Relationship between breeding success and size

**Figure 3.**
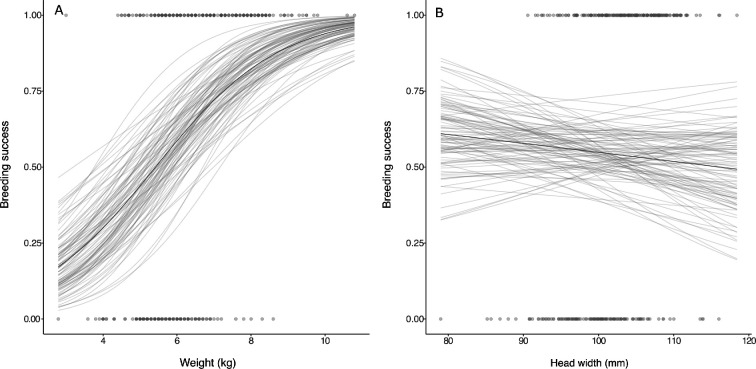
(A) Plot showing the relationship between body weight with annual breeding success and (B) between head width and annual breeding success Points show observed data and regression lines show the predicted relationship between size traits and annual breeding success derived from a mixed effects model that fits annual breeding success as a function of both size traits (see §2 for full model structure). Solid dark lines show predictions derived from the median of the posterior and the lighter lines show 100 randomly selected draws from the posterior.

We did not find evidence that head width had an effect on annual breeding success in female Tasmanian devils (table 1, figure 3 and electronic supplementary material, figure S3). However, heavier females were more likely to breed than those with lower body weight (table 1 and figure 3). We did not find evidence for an interaction between age and body weight (table 1 and electronic supplementary material, figure S3), suggesting that the pattern of heavier females being more likely to breed was consistent across 1-year-olds and adults. We did find evidence for an interaction between head width and age (table 1). However, this significant interaction seems unlikely to be biologically meaningful as the main effect for head width was not different from zero, and the cross-over effect that this interaction suggests does not appear until the larger values of head width measurements (electronic supplementary material, figure S1), where there are fewer data and therefore a great amount of uncertainty. There was no evidence for a relationship between having DFTD and likelihood of breeding in a given year (table 1), suggesting that being infected with DFTD did not affect the probability of females breeding.

### (c) Inbreeding depression

We did not find evidence for a relationship between annual breeding success and the individual inbreeding coefficient, *F*_GRM,_ either for 1-year-olds or for adults (table 2 and electronic supplementary material, figure S4), suggesting that there was little to no evidence for inbreeding depression in the population, even when estimated for each age category separately. This was true when we fit the full model, or fit a reduced model with just age and *F*_GRM_ fit as fixed effects, suggesting that removing the fixed effects with little statistical support in the previous model did not affect our estimates of the effect of inbreeding.

**Table 2.** Table summarizing results from a mixed effects model used to estimate inbreeding depression on annual breeding success. The model was run as a logistic regression given that annual breeding success is a binary variable, and therefore all parameters are presented on the logit scale. Posterior medians of linear coefficient estimate for fixed effects and standard deviations for random effects are presented with 95% credible intervals of posterior distribution in parentheses. Full posteriors can be found in the electronic supplementary material.

fixed effects	age_ADULT_	7.76 (3.24 to 13.62)
	head width	0.24 (−0.27 to 1.00)
	weight	4.76 (1.87 to 9.31)
	*F*_GRM_	1.64 (−7.45 to 10.45)
	age_ADULT_: *F*_GRM_	−1.04 (−10.72 to 8.65)
random effects	ID	5.61 (0.90 to 12.97)
	trapID	2.78 (0.13 to 7.61)
	year	1.37 (0.04 to 4.60)
	month	1.65 (0.06 to 5.47)

Age was fit as a two-level factor (‘Adult’ or ‘1-year-old’), where the reference level was 1-year-old females; head width (mm) and weight were fit as linear effects; *F*_GRM_ was fit as a linear effect and describes the effect of the genetic inbreeding coefficient on annual breeding success; Age_ADULT_: *F*_GRM_ describes the interaction between age and inbreeding depression; ID was the unique identifier of each female and accounted for repeated measures of females; trapID was the unique identifier of each location and accounted for spatial environmental variation; random effect of year accounted for nonlinear changes in probability of breeding; month accounted for seasonality in breeding.

## 4. Discussion

We analysed data from a long-term mark–recapture study of a wild, declining population of Tasmanian devils to test hypotheses related to observations of disease-induced female precocial breeding detected 20 years ago [15]. **After** prior observations that age structure in populations with high DFTD prevalence collapsed [32] and that adult females (2 years old) became semelparous**, w**e tested whether this life-history shift, representing a classic trade-off between growth, survival and reproduction, continued over time. Additionally, we tested for evidence of inbreeding depression, as population sizes collapsed by more than 80% in most infected devil populations [26,28]. With 15 years of additional data, we found that there was no evidence for a continued trend in increased probability of 1-year-olds breeding, and the probability of 1-year-olds breeding has plateaued at a rate of about 40% for the past 18 years. We also found that while heavier females were more likely to breed overall, there was no evidence for a differential effect of size on breeding success for 1-year-olds or individuals that were over 2 years old. Finally, we found that breeding success was not under inbreeding depression for either age category.

According to life-history theory, early reproduction should be selected for when population perturbances impact adult survival more than juvenile survival [41]. DFTD almost exclusively impacts survival in adult Tasmanian devils, as the youngest age when a devil may contract the disease is around 14 months [15]. The original rise in rates of precocial breeding found immediately after the emergence of DFTD [15] may have reflected either phenotypic plasticity in timing of reproduction or selection induced by DFTD. Our results suggest that, despite the early increase in the probability of 1-year-olds breeding, the trend did not continue post-2003. This result suggests that the initial response in precocial breeding may have reflected plasticity rather than selection because we would have expected a consistent linear trend if DFTD (which continued to increase in prevalence and cause high rates of mortality and population declines) was causing directional selection. Alternatively, the population may have faced constraints that inhibited a further increase in the proportion of precocial breeders. Such constraints could be caused, for instance, by limits imposed on individual growth rates in the population. That is, females that breed precocially normally breed in May, which is late in the breeding season [28], and their female offspring cannot then grow enough to reach maturity upon weaning and den departure by May of the subsequent year, resulting in inhibition of precocial breeding. An additional constraint could be caused by having a mother with DFTD, which would compromise the nutrition of her young as tumours grew [42]. A promising avenue of future research would be to test this hypothesis once the necessary data are available.

Body weight is often positively correlated with reproductive success, owing to enhanced ability to provision resources to offspring [43]. Accordingly, body weight is often found to be under selection in populations of wild animals [44]. Here, we found that heavier female Tasmanian devils were more likely to breed, and this effect did not differbetween age classes. Thus, body weight may be under selection in this population, and based on this result alone, we might expect body weight to increase over time. Interestingly, this prediction is further supported by work showing that heavier individuals are also less likely to contract DFTD [40]. However, in the population studied here, mean body weight has not changed since 1999 [40]. A lack of temporal change despite evidence for positive selection has been found in other systems [45,46] and may be owing to, for instance, a fluctuating environment that can alter the selective landscape or covariances with other unmeasured traits [47–49]. However, such a ‘paradox-of-stasis’ remains a major unanswered question in evolutionary biology and empirical research is needed to test hypotheses that may explain how this phenomenon emerges [50].

Inbreeding depression, defined as a negative correlation between homozygosity and fitness [20], can be a serious risk to declining populations [18,21] and can affect life-stages differently [23–25]. We found no evidence for inbreeding depression on breeding success in either adult or subadult females. These results are surprising in light of previous studies showing that genetic diversity is quite low in devils across their species range [51]. A possible explanation is that deleterious alleles have been purged from the population during the observed population decline [52]. Indeed, previous work has shown sufficient genetic diversity for devils to respond to selection [34,35,53–55], as well as a lack of evidence for inbreeding depression in susceptibility to DFTD [40]. However, note that inbreeding depression is highly dependent on present and future environmental conditions, and may be present in other fitness-related traits not measured here (e.g. survival). Therefore, conclusions about the demographic consequences of inbreeding in Tasmanian devils should not be drawn from our results alone.

It is important to note that the lack of a relationship reported here between inbreeding, size and breeding success may not reflect patterns in Tasmanian devil populations outside the Freycinet peninsula, and there remains the possibility that inbreeding depression in annual breeding success or changes in breeding success through time are occurring in other populations. However, although ‘null-results’ should not be over-interpreted as the absence of an effect, our work shows that, despite an initial rise in the proportion of females precocially breeding after the emergence of DFTD, the probability of 1-year-old females breeding has not continued to rise thereafter. Our work thus demonstrates the value of long-term, multigenerational population studies [56,57] and the importance of follow-up studies when trying to understand temporal changes in life-history traits. Finally, important questions remain unanswered here about the underlying mechanisms associated with the initial rise of precocial breeding followed by the plateau that we observed. Determining the extent to whichthose changes were associated with selection or plasticity, as well as tracking any temporal changes in growth rates, could ultimately provide key insights as to how life-history strategies may respond to population perturbances.

## Data Availability

All data and code needed to reproduce results in this manuscript have been archived in a Zenodo repository [58]. Supplementary material is available online [59].
